# Antibiotic stewardship in non-perforated gangrenous appendicitis: outcomes of single-dose versus postoperative therapy in a retrospective cohort study

**DOI:** 10.1007/s00423-026-04129-9

**Published:** 2026-07-03

**Authors:** Cihan Ozen, Ali Yalcinkaya, Maiken Cecilie Eberhard, Annette Geest Soerensen, Bade Ozen, Anders Oest, Deniz Karasoy, Peter Derek Christian Leutscher, Kathrine Holte

**Affiliations:** 1 Department of Gastrointestinal Surgery, Aalborg University Hospital, Aalborg, Denmark; 2 Department of Clinical Medicine, Aalborg University Hospital, Aalborg, Denmark; 3 Interdisciplinary Orthopaedics, Aalborg University Hospital, Aalborg, Denmark; 4 Faculty of Medicine, Aalborg University Hospital, Aalborg, Denmark; 5 Faculty of Medicine, San Raffaele University, Milan, Italy; 6 Institute of Surgical Pathology, North Denmark Regional Hospital, Hjoerring, Denmark; 7 Department of Medicine, Holbaek Hospital, Medicine 1, Holbaek, Denmark; 8 Centre for Clinical Research, North Denmark Regional Hospital, Hjoerring, Denmark; 9 Department of Gastrointestinal Surgery, North Denmark Regional Hospital, Hjoerring, Denmark

**Keywords:** Non-perforated gangrenous appendicitis, Postoperative antibiotic therapy, Postoperative surgical complications, Intra-abdominal abscess, Length of hospital stay

## Abstract

**Purpose:**

Nonperforated gangrenous appendicitis (NGA) is a severe inflammatory condition without perforation, and the optimal duration of postoperative antibiotic therapy remains debated. This study aimed to compare outcomes between single-dose antibiotic therapy (SDAT) and postoperative antibiotic therapy (POAT).

**Methods:**

This retrospective study compared outcomes among 242 adult patients diagnosed histopathologically with NGA after laparoscopic appendectomy who received SDAT versus POAT antibiotics. Demographic characteristics, clinical scores, inflammatory biomarkers, antibiotic regimens, and postoperative surgical infectious complications (PSCs) within 30 days were evaluated. To address group non-homogeneity, regression analyses were performed, and propensity score matching (PSM) was applied as a sensitivity analysis.

**Results:**

Among the cohort, 68.2% received SDAT and 31.8% received POAT. In the unadjusted analysis, SDAT was associated with significantly shorter hospital stays (14 vs. 79 h; *p* < 0.001), and PSC rates were lower in the SDAT group (3.6% vs. 11.7%; *p* = 0.022). However, POAT patients had higher baseline severity markers, older age, elevated CRP, and higher ASA and q-SOFA scores. After propensity score matching on these variables, PSC rates were comparable between groups (11.3% vs. 9.7%; OR 0.84, 95% CI 0.27–2.66; *p* = 0.769), with no significant differences in intra-abdominal abscess or surgical site infection rates.

**Conclusions:**

These findings suggest that SDAT is a sufficient antibiotic strategy for non-perforated gangrenous appendicitis and supports its use as routine practice in line with antibiotic stewardship principles. However, given the retrospective design, causal inference is limited, and prospective multicentre studies are warranted to confirm these findings.

**Supplementary Information:**

The online version contains supplementary material available at https://doi.org/10.1007/s00423-026-04129-9.

## Introduction

Acute appendicitis is the second most common cause of abdominal pain among patients admitted to surgical emergency departments [1]. Diagnosis is typically established through a progression of clinical assessments by the surgeon, intraoperative macroscopic evaluation, and postoperative histopathological examination. Based on these findings, acute appendicitis is commonly classified into three distinct categories: phlegmonous appendicitis, non-perforated gangrenous appendicitis (NGA), and appendicitis with perforation or abscess [2].

A multicentre study involving 3,597 patients found that 7% were histopathologically diagnosed with gangrenous appendicitis. Among 219 clinically confirmed cases, 26% were phlegmonous, 53% gangrenous, and 11% perforated [3].

Current evidence suggests that patients with phlegmonous appendicitis do not require extended postoperative antibiotic therapy [4–6]. In contrast, patients with complicated appendicitis involving perforation and/or abscess typically receive antibiotics for approximately 3–5 days following surgery [7–9]. However, the optimal treatment approach for NGA remains controversial [8]. Recent multicentre data suggest that gangrenous appendicitis represents a clinically recognizable severity phenotype, often influencing perioperative decision-making. A large study by Liang et al. [10] showed that laboratory and CT findings can reliably identify gangrenous or perforated disease preoperatively, underscoring why antibiotic strategies may vary among patients.

In clinical practice, patients with NGA are generally treated with either a single-dose antibiotic therapy (SDAT) or a postoperative antibiotic therapy (POAT) regimen, with the choice often based on the perceived clinical severity [11, 12]. This severity classification is essential for estimating the risk of postoperative surgical infectious complications (PSCs), which include intra-abdominal abscess (IAA), sepsis, peritonitis, and surgical site infections (SSIs) [13]. In this study, PSCs were strictly defined as intra-abdominal abscess (IAA) or surgical site infections (SSI) occurring within 30 days.

Postoperative complications following laparoscopic appendectomy are generally low, with reoperation rarely required [11, 14]. Specifically, for patients with non-perforated gangrenous appendicitis, the literature reports an overall PSCs incidence of 7.2% [15]; with postoperative IAA occurring in 4.7% [14], and SSIs in 2.8% [16] of cases.

This study aimed to investigate whether there is a difference in the incidence of postoperative surgical infectious complications for NGA, such as intra-abdominal abscesses and surgical site infections within 30 days, between patients receiving single-dose antibiotic therapy and those receiving postoperative antibiotic therapy.

## Methods

### Study design

This retrospective clinical quality assurance study was conducted by reviewing the medical records of patients who underwent surgery for acute appendicitis at the North Denmark Regional Hospital between March 2018 and December 2021. The diagnosis of non-perforated gangrenous appendicitis (NGA) was confirmed by histopathological examination.

### Study population

**Fig. 1:**
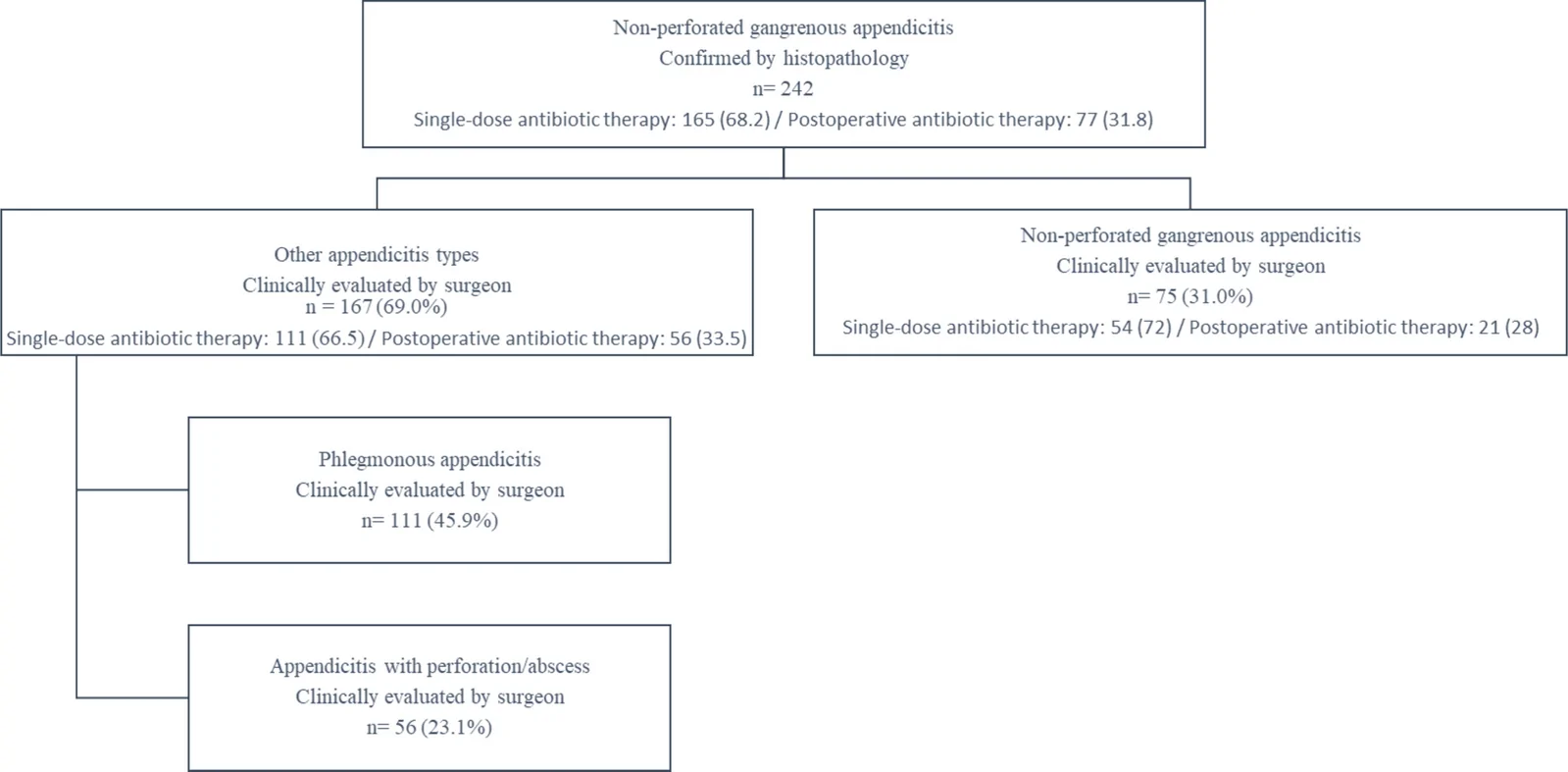
Flowchart of all histopathologically confirmed non-perforated gangrenous appendicitis after laparoscopic appendectomy

Data were collected from 242 adult patients with histopathologically confirmed NGA who underwent laparoscopic appendectomy during the study period. Among these patients, clinicians perioperatively evaluated phlegmonous appendicitis in 111 (45.9%), NGA in 75 (31.0%), and appendicitis with perforation or abscess in 56 (23.1%) cases (Fig. 1). For clarity, the exposure groups (SDAT vs. POAT) were defined within the full histopathology-confirmed NGA cohort (*n* = 242), not only within the 75 cases that were also clinically classified as NGA intraoperatively. Hence, counts such as SDAT = 165 and POAT = 77 refer to the histopathology-confirmed NGA universe.

### Population inclusion and exclusion criteria

The study included adult patients (age ≥ 18 years) with a histopathologically confirmed diagnosis of NGA (*n* = 242). Eighteen patients were excluded because they were under 18 years of age or had incomplete or inconclusive data records.

### Inflammatory biomarkers

Blood samples for routine biochemical and hematological analyses [17], including leukocyte count (LC), neutrophil count (NC), and C-reactive protein (CRP), were obtained within two hours of admission to the surgical emergency department. Laboratory results were available for clinical evaluation within one hour. The reference values for the inflammatory biomarkers were: LC (≥ 10.0 × 10⁹/L), NC (≥ 7.00 × 10⁹/L), and CRP (≥ 8.0 mg/L). The Neutrophil-to-Leukocyte Ratio (NLR) [18] was calculated by dividing the absolute neutrophil count by the total leukocyte count. The derived Neutrophil-to-Lymphocyte Ratio (d-NLR) was calculated by dividing the absolute neutrophil count by the difference between total leukocytes and neutrophils [19].

### Antibiotic treatment

All patients received preoperative antibiotic prophylaxis. The regimens included:

- IV cefuroxime 1500 mg (750 mg every 6 h) and IV metronidazole 1000 mg (500 mg every 12 h),
- IV benzylpenicillin 5 million IU (1.2 million IU every 6 h) with gentamicin 5 mg/kg and IV metronidazole 1000 mg (500 mg every 12 h), or
- IV piperacillin 4 g + tazobactam 0.5 g (every 6 h).

According to regional antibiotic guidelines, patients with NGA received a single prophylactic antibiotic dose 30 min before laparoscopic appendectomy. However, based on clinical evaluation, some patients received a three-day postoperative antibiotic course, depending on clinical evaluation by the surgeon. Operational exposure definitions were as follows: Single-dose antibiotic therapy (SDAT) indicated preoperative prophylaxis only, administered approximately 30 min before incision. Postoperative antibiotic therapy (POAT) indicated continuation of systemic antibiotics for ≥ 24 h beyond the induction dose, typically for 48–72 h at the surgeon’s discretion.

### Study data

Data were collected and managed using RedCap (Research Electronic Data Capture) tools hosted at Region North Hospital [20]. Redcap is a secure, web-based platform designed to support data capture for research studies.

For each of the 242 patients with histopathologically confirmed NGA, the following data were retrieved from the electronic patient records; age, gender, ASA (American Society of Anaesthesiologists) score [21], q-SOFA (quick sequential organ failure assessment) score [22], clinical diagnosis, Alvarado score [23], biomarkers (LC, NC, CRP, NLR, d-NLR), antibiotic regime and treatment duration, operations delay, length of hospital stay, and postoperative surgical infectious complications (PSCs) were stringently defined as the occurrence of either intra-abdominal abscesses (IAA) or surgical site infections (SSI) within 30 days following the surgical procedure.

### Pathological diagnosis

Following laparoscopic appendectomy, the excised appendix was preserved in 4% formalin for histopathological examination. An experienced pathologist classified the findings as phlegmonous appendicitis, non-perforated gangrenous appendicitis (NGA), or appendicitis with perforation or abscess [24].

The diagnosis of gangrenous appendicitis was based on the severity of inflammation, as determined by histological examination. Gangrenous appendicitis was characterized by inflammatory cell infiltration and structural tissue damage, without abscess formation or perforation [2].

### Ethical considerations and project registration

The study was registered in the North Denmark Region clinical research registry under ID number K2023-024. As a retrospective clinical quality assurance study, approval from the Scientific Ethics Committee for Region North Jutland has confirmed that no ethical approval is required.

### Statistical analysis

Data were analyzed according to clinical severity and antibiotic regimen. Categorical variables were compared using the Pearson chi-square test or Fisher’s exact test, as appropriate. Continuous variables were assessed for normality using the Kolmogorov–Smirnov test. Normally distributed variables are presented as mean ± standard deviation (SD) with 95% confidence intervals (CIs) and were compared using the independent-samples t test. Non-normally distributed variables are reported as median and interquartile range (IQR) and were compared using the Mann–Whitney U test. A two-sided *p* value of < 0.05 was considered statistically significant. All analyses were performed using IBM SPSS Statistics, version 27.0.

To address potential bias arising from group non-homogeneity, two complementary analytical approaches were undertaken. First, associations between clinical variables, antibiotic regimen, and postoperative surgical infectious complications (PSCs) were evaluated using univariable and multivariable logistic regression analyses. Variables with a *p* value < 0.20 in the univariable analysis, together with clinically relevant covariates identified a priori, were considered for inclusion in the multivariable model. In the multivariable logistic regression model, POAT was coded as the reference category; thus, odds ratios < 1 indicate a higher likelihood of receiving POAT with increasing predictor values.

Second, propensity score matching (PSM) was performed as a pre-specified sensitivity analysis using nearest-neighbour matching with a caliper width of 0.1. Matching was based on age, CRP level, ASA score, and q-SOFA score. Covariate balance before and after matching was assessed using standardized mean differences. Within the matched cohort, PSC rates were compared between the SDAT and POAT groups.

## Results

### Patient cohort

Out of 242 patients with histopathologically confirmed non-perforated gangrenous appendicitis (NGA), 75 (31%) were also clinically diagnosed with NGA during surgery (Fig. 1). Within the histopathologically confirmed NGA cohort, 165 (68.2%) received SDAT, and 77 (31.8%) received POAT; in the clinical and histopathology NGA subset (*n* = 75), 54 (72%) received SDAT, and 21 (28%) received POAT.

### Demographics and clinical characteristics

The median age was significantly higher in the POAT group compared to the SDAT group (52 vs. 38 years, *p* < 0.001). Gender distribution and clinical scores (ASA, q-SOFA, Alvarado) were similar across groups. Most patients had low ASA and q-SOFA scores (Table 1).

**Table 1:** Outcomes of postoperative complications and antibiotic therapy in patients with non-perforated gangrenous appendicitis

		Antibiotic Regimen		*P*-value
*Histopathologically confirmed NGA,* n (%) = 242		SDAT	POAT	
		165 (68.2)	77 (31.8)	
Age, median (IQR)	43 (28–59)	38 (26–54)	52 (39–65)	* < 0.001
Gender	Female; 123 (50.8)/Male;119 (49.2)	85 (51.5)/80 (48.5)	38 (49.4)/39 (50.6)	**0.754
ASA Score	I – II; 206 (85.1)/III; 36 (14.9)	148 (89.7)/17 (10.3)	58 (75.3)/19 (24.7)	**0.003
q-SOFA Score	Score 0; 222 (91.7)/Score 1; 20 (8.3)	159 (94.5)/9 (5.5)	66 (85.7)/11 (14.3)	**0.020
Alvarado Score, median (IQR)	8 (7–9)	8 (8–9)	8 (6–9)	*0.608
Biomarkers (admission)	CRP (mg/L), median (IQR), 45 (19–79)	36 (15–69)	70 (31–115)	* < 0.001
	LC (10^9^/L), mean ± SD (95% CI: 13.2 ± 3.7 (12.7–13.7)	13.2 ± 3.6 (12.7–13.8)	13.1 ± 4.0 (12.2–14.1)	****0.879
	NC (10^9^/L), mean ± SD (95% CI): 10.7 ± 3.5 (10.2–11.1)	10.7 ± 3.3 (10.2–11.2)	10.8 ± 3.7 (9.9–11.6)	****0.823
	NC/LC Rate (%), median (IQR); 81 (76–87)	81 (76–85)	82 (76–88)	*0.289
	d-NLR, median (IQR); 4.15 (3.11–6.20)	4.10 (3.11–5.65)	4.44 (3.11–6.85)	*0.302
Operations delay (hours), median (IQR)	6.5 (4–11)	7 (4–11)	6 (4–11)	*0.874
Length of hospital stay (hours), median (IQR)	17 (13–65)	14 (11–17)	79 (67–95)	* < 0.001
Antibiotic regimen	Cefuroxime + metronidazole, *n* = 163 (67.4)	119 (72.1)	44 (57.1)	***0.004
	Benzylpenicillin/gentamicin + metronidazole, *n* = 70 (28.9)	44 (26.7)	26 (33.8)	
	Piperacillin and tazobactam, *n* = 9 (3.7)	2 (1.2)	7 (9.1)	
No complications	*n* = 227 (93.8)	159 (96.4)	68 (88.3)	***0.022
Postoperative surgical infectious complications	*n* = 15 (6.2)	6 (3.6)	9 (11.7)	
	Intra-abdominal abscess, *n* = 7 (2.9)	1 (16.7)	6 (66.7)	***0.119
	Surgical site infections, *n* = 8 (3.3)	5 (83.3)	3 (33.3)	
		Antibiotic Regimen		P-value
*Clinical and histopathologically confirmed NGA*, *n* = 75		SDAT	POAT	
		54 (72)	21 (28)	
No complications	*n* = 71 (94.7)	51 (94.4)	20 (95.2)	***1.000
Postoperative surgical infectious complications	*n* = 4 (5.3)	3 (5.6)	1 (4.8)	
	Intra-abdominal abscess, *n* = 1 (1.3)	0 (-)	1 (100)	***0.250
	Surgical site infections, *n* = 3 (4.0)	3 (100)	0 (-)	

IQR: 25th–75th Interquartile range. Abbreviations: *NGA* non-perforated gangrenous appendicitis, *SDAT* Single-dose antibiotic therapy, *POAT* postoperative antibiotic therapy, *ASA* American Society of Anesthesiologists, *q-SOFA* quick Sepsis related Organ Failure Assessment, *SD* Standard deviation, *CI* Confidence interval, *LC* leukocyte count, *NC* neutrophil count, *CRP* C-reactive protein, *d-NLR* derived Neutrophil-to-Lymphocyte Ratio

^*^Mann Whitney U test (No normal distribution) **Pearson Chi-square test *** Fisher’s Exact test ****Independent T-test (Normal distribution)

### Antibiotic regimens and surgical timing

Cefuroxime with metronidazole was the most commonly used antibiotic. Single-dose antibiotic therapy (SDAT) was administered to most patients. Operation delay was comparable between groups (Table 1).

### Postoperative antibiotic therapy (POAT)

POAT was administered to 31.8% of patients in the histopathological group and 28% in the combined clinical and histopathological group, primarily based on the surgeon’s clinical evaluation of disease severity. In the histopathological group, POAT was significantly associated with older age, higher ASA and q-SOFA scores, and elevated CRP levels. In the combined clinical and histopathological group, only the ASA score showed a significant association***.*** POAT patients had significantly longer hospital stays in both cohorts (Table 1).

### Antibiotic regimen differences

SDAT patients were more likely to receive cefuroxime with metronidazole, while POAT patients more often received broader-spectrum antibiotics such as piperacillin/tazobactam (Table 1).

### Postoperative surgical infectious complications (PSCs)

PSC rates were 6.2% in the histopathological group and 5.3% in the combined clinical and histopathological group. PSCs were significantly more frequent in POAT patients. Intra-abdominal abscesses were more frequent in POAT patients, whereas surgical site infections were slightly more common in SDAT patients; however, neither difference reached statistical significance (Table 1).

### Comparison between patients with and without PSCs

Clinical and laboratory parameters did not differ significantly between patients with and without PSCs. However, patients without PSCs were more likely to have received single-dose antibiotic therapy (SDAT) and had significantly shorter hospital stays (Table 2).

**Table 2:** Comparison between postoperative complications and no complication non-perforated gangrenous appendicitis

*Histopathologically confirmed NGA, n (%)* = *242*		PSCs	No complications	P-Value
		15 (6.2)	227 (93.8)	
Age, median (IQR)		38 (24–62)	43 (29–59)	*0.833
Gender (Female/Male)		9 (60)/6 (40)	114 (50)/113 (50)	**0.463
ASA Score (I-II/III)		12 (80)/3 (20)	194 (86)/33 (14)	***0.474
q-SOFA Score (Score 0/Score 1)		12 (80)/3 (20)	210 (93)/17 (7)	***0.115
Alvarado Score, median (IQR)		9 (8–9)	8 (7–9)	*0.530
Biomarkers (admission)	CRP (mg/L), median (IQR)	54 (28–72)	45 (18–80)	*0.696
	LC (10^9^/L) ± SD, mean (95% CI)	13.6 ± 3.2 (11.8–15.3)	13.2 ± 3.7 (12.7–13.6)	****0.676
	NC (10^9^/L) ± SD, mean (95% CI)	11.2 ± 2.9 (9.6–12.8)	10.7 ± 3.5 (10.2–11.1)	****0.546
	NC/LC Rate (%), median (IQR)	81 (77–89)	81 (76–87)	*0.321
	d-NLR, median (IQR)	4.1 (3.3–7.8)	4.2 (3.1–6.2)	*0.353
Operations delay (hours), median (IQR)		7 (4–15)	6 (4–11)	*0.388
Antibiotic treatment	Cefuroxime	10 (67)	153 (67)	***0.145
	Benzylpenicillin/gentamycin	3 (20)	67 (30)	
	Piperacillin and tazobactam	2 (13)	7 (3)	
Antibiotic regime	SDAT	6 (40)	159 (70)	***0.022
	POAT	9 (60)	68 (30)	
Length of hospital stay (day), median (IQR)		4 (1–5)	1 (1–3)	*0.003
*Clinical and histopathologically confirmed NGA, n (%)* = *75*		PSC	No complications	P-Value
		4 (5.3)	71 (94.7)	
Antibiotic regime	SDAT	3 (75)	51 (72)	***1.000
	POAT	1 (25)	20 (28)	
Length of hospital stay (day), median (IQR)		1 (1–2)	1 (1–3)	*0.928

IQR:25th–75th Interquartile range. Abbreviations: *NGA* Non-perforated gangrenous appendicitis, *PSCs* Postoperative surgical infectious complications, *ASA* American Society of Anesthesiologists, *q-SOFA* quick Sepsis related Organ Failure Assessment, *SD* Standard deviation, *CI* Confidence interval, *LC* leukocyte count, *NC* neutrophil count, *CRP* C-reactive protein, *d-NLR* derived Neutrophil-to-Lymphocyte Ratio, *SDAT* single-dose antibiotic therapy, *POAT* postoperative antibiotic therapy

^*^Mann Whitney U test ((No normal distribution) **Pearson Chi-Square test ***Fisher’s Exact test ****Independent T-test (Normal distribution)

### Univariable and multivariable logistic regression analyses

In the univariable logistic regression analysis, POAT was associated with higher odds of postoperative surgical infectious complications (PSCs) compared with SDAT (OR 3.51, 95% CI 1.20–10.24; *p* = 0.022). None of the other evaluated clinical or laboratory variables, including age, sex, ASA score, q-SOFA score, Alvarado score, operation delay, CRP level, leukocyte count, neutrophil count, or d-NLR, were significantly associated with PSCs.

In the multivariable logistic regression analysis, POAT remained independently associated with increased odds of PSCs (OR 3.38, 95% CI 1.07–10.67; *p* = 0.037), whereas no other variable reached statistical significance (Table 3). Given the limited number of outcome events (*n* = 15) and the resulting wide confidence intervals, these estimates should be interpreted with caution and considered in conjunction with the propensity score–matched analysis.

**Table 3:** Univariate and Multivariable Logistic Regression Analyses of Postoperative Surgical Complications in Non-Perforated Gangrenous Appendicitis

Postoperative surgical infectious complications, *n* = 15 (6.2)	Histopathologically confirmed NGA, n (%) = 242			
	Univariate		Multivariate	
	OR (95% CI)	P-value	OR (95% CI)	P-value
Age, years	1.00 (0.97–1.03)	0.987	0.98 (0.94–1.02)	0.231
Sex (Male)	1.49 (0.50–4.40)	0.466	-	
ASA Score (per 1-point increase)	1.68 (0.85–3.33)	0.138	1.92 (0.81–4.56)	0.141
q-SOFA Score (0 vs ≥ 1)	3.09 (0.79–12.01)	0.104	1.98 (0.47–8.23)	0.352
Alvarado Score (per 1-point increase)	1.11 (0.80–1.53)	0.542	1.11 (0.79–1.55)	0.554
Antibiotic Treatment, n (%)				
Cefuroxime + metronidazole	Reference 0.69 (0.18–2.57)	-	-	-
Benzylpenicillin/gentamycin + metronidazole	0.69 (0.18–2.57)	0.575	-	-
Piperacillin and tazobactam	4.37 (0.80–23.85)	0.088	-	-
Antibiotic regimen (POAT vs SDAT)	3.51 (1.20–10.24)	**0.022**	3.38 (1.07–10.67)	**0.037**
Operations delay (hour) (per 1-h increase)	1.05 (0.96–1.14)	0.288	-	-
Biomarkers (admission)				
CRP (mg/L)	1.00 (0.99–1.01)	0.813	-	-
LC (× 10^9^/L)	0.72 (0.21–2.43)	0.593	-	-
NC (× 10^9^/L)	1.51 (0.36–6.27)	0.573	-	-
d-NLR	0.96 (0.64–1.45)	0.859	-	-

Abbreviations: *NGA* Non-perforated gangrenous appendicitis, *ASA* American Society of Anesthesiologists, *q-SOFA* quick Sepsis-related Organ Failure Assessment, *LC* leukocyte count, *NC* neutrophil count, *CRP* C-reactive protein, *d-NLR* derived Neutrophil-to-Lymphocyte Ratio, *SDAT* single-dose antibiotic therapy, *POAT* postoperative antibiotic therapy, *LOS* Length of hospital stay, *OR* odds ratio

### Propensity score–matched sensitivity analysis

**Fig. 2:**
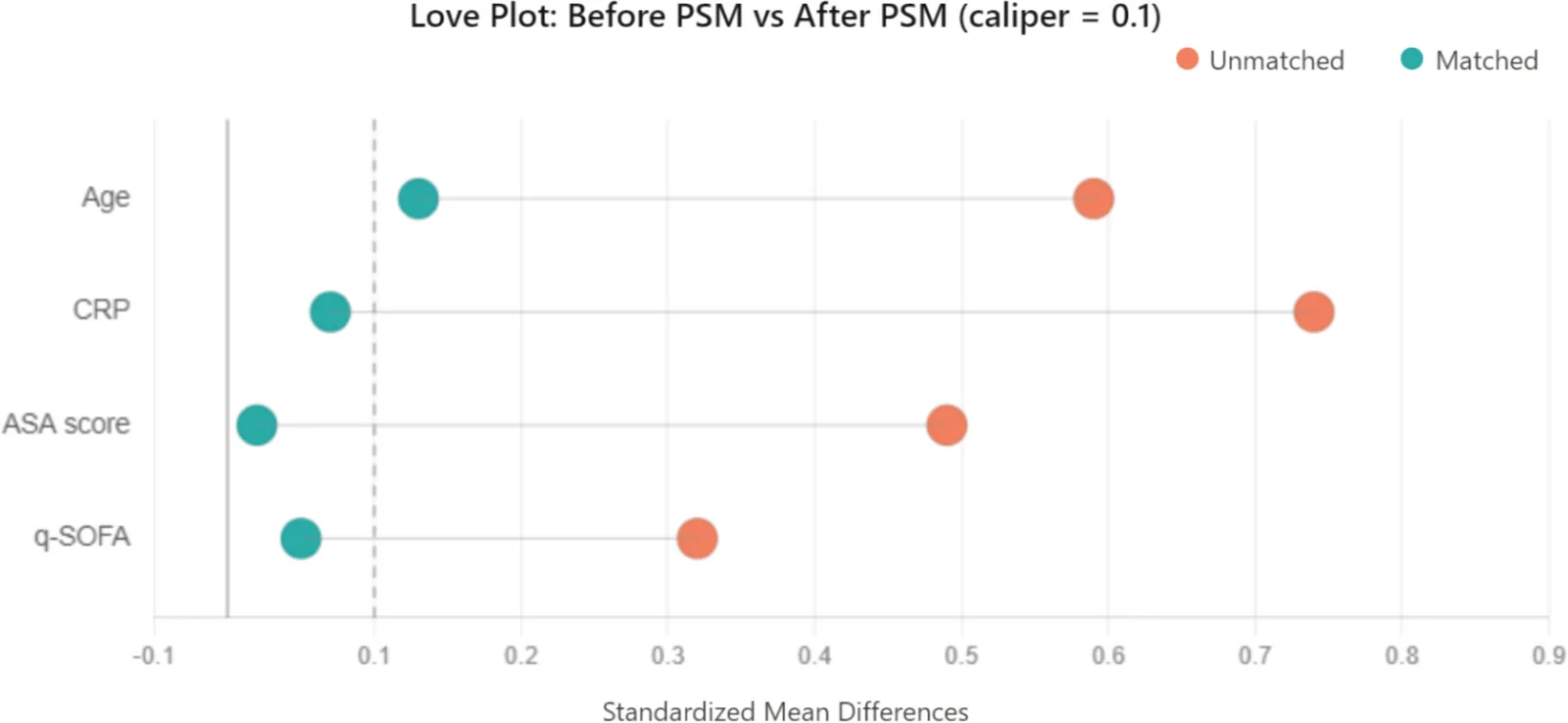
Love plot showing standardized mean differences of baseline covariates before and after propensity score matching (caliper = 0.1). Covariate balance was considered adequate at standardized mean differences < 0.1

Because of the potential for confounding by indication in the regression analyses, propensity score matching was performed as a sensitivity analysis to account for baseline differences between antibiotic-regimen groups. Matching based on age, CRP level, ASA score, and q-SOFA score yielded 62 matched pairs using a caliper width of 0.1. Adequate covariate balance was achieved after matching, as demonstrated by standardized mean differences (Fig. 2). Within the matched cohort, PSCs occurred in 11.3% of patients treated with SDAT and 9.7% of those receiving POAT (OR 0.84, 95% CI 0.27–2.66; *p* = 0.769). Rates of intra-abdominal abscess and surgical site infection were similarly comparable between the two groups (Table 4).

**Table 4:** Propensity Score Matching (PSM) Sensitivity Analysis Results (Caliper = 0.1, 62 matched pairs)

PSM: SDAT vs POAT	Reference (SDAT)	Exposed (POAT)	OR (95% CI)	P-value
Postoperative surgical infectious complications (PSCs), n (%)	7 (11.3)	6 (9.7)	0.84 (0.27–2.66)	*0.769
└Intra-abdominal abscess (IAA), n (%)	4 (6.5)	1 (1.6)	0.24 (0.03–2.19)	**0.365
└Surgical site infection (SSI), n (%)	3 (4.8)	5 (8.1)	1.73 (0.39–7.56)	**0.717

PSM: Propensity score matching based on age, CRP level, ASA score (numeric), and q-SOFA score (numeric). PSCs was defined as a composite outcome consisting of intra-abdominal abscess and/or surgical site infection. Outcome rates are reported descriptively in the matched cohort

In the propensity score–matched cohort using a caliper of 0.1, postoperative surgical infectious complications occurred in 11.3% of patients treated with SDAT and 9.7% of those receiving POAT, with no meaningful difference observed between treatment strategies

^*^Pearson Chi-Square test **Fisher’s Exact test

To further assess potential selection bias in antibiotic prescribing, factors associated with the use of postoperative antibiotic therapy were evaluated using a multivariable logistic regression model (Supplementary Table S1). Age, CRP level, and q-SOFA score were independently associated with POAT use. This reflects the way the model was specified rather than a true inverse clinical relationship.

## Discussion

In this single-centre retrospective cohort, the initial unadjusted analysis suggested higher rates of postoperative surgical infectious complications and longer hospital stay among patients receiving postoperative antibiotic therapy (POAT) compared with those treated with single-dose antibiotic therapy (SDAT). However, this finding should be interpreted in the context of baseline clinical differences between groups. Patients selected for POAT were older and had higher CRP, ASA, and q-SOFA scores, indicating that extended antibiotic therapy was preferentially prescribed to patients perceived as having greater clinical severity.

To address this imbalance, propensity score matching was performed using age, CRP level, ASA score, and q-SOFA score. After matching, 62 balanced pairs were obtained, and postoperative infectious complication rates were comparable between the SDAT and POAT groups, with no significant differences in intra-abdominal abscess or surgical site infection rates. Notably, the difference observed in the unadjusted analysis was no longer present after adjustment for baseline severity. These findings suggest that routine postoperative antibiotic therapy may not necessarily provide additional benefit over SDAT in patients with non-perforated gangrenous appendicitis and support SDAT as a sufficient treatment strategy in this clinical setting. Importantly, these results should be interpreted as evidence of non-inferiority rather than superiority of SDAT.

Our results are consistent with the broader literature on antibiotic stewardship in appendicitis. The meta-analysis by Kurdi et al. [25] found no added benefit of routine postoperative antibiotics in uncomplicated appendicitis and emphasized antimicrobial stewardship. In a national multicentre cohort of non-perforated gangrenous cases, de Wijkerslooth et al. [15] reported that > 24 h of postoperative antibiotics did not reduce infectious complications compared with ≤ 24 h, questioning prolonged regimens in this subgroup, though a trend toward more complications with longer treatment was noted. While Liang et al. [10] showed that CT and laboratory markers can help predict gangrenous or perforated disease preoperatively, our findings caution against automatically translating the recognition of gangrenous inflammation into an indication for extended antibiotic therapy when intraoperative findings are reassuring and postoperative recovery is uncomplicated.

The significant reduction in hospital stay observed in the SDAT group (14 vs. 79 h; *p* < 0.001) is clinically meaningful and consistent with published evidence. Nordin et al. [11] and Devin et al. [26] both demonstrated that shorter antibiotic regimens were associated with reduced hospitalisation duration and resource utilisation without increasing complication rates. These findings collectively support the reclassification of non-perforated gangrenous appendicitis as uncomplicated appendicitis for the purpose of antibiotic prescribing, and the adoption of single-dose perioperative prophylaxis as standard practice in line with antibiotic stewardship principles and Danish national guidelines [27].

Our findings highlight the value of standardized histopathological confirmation as a guide to treatment decisions. In our cohort, a substantial proportion of patients clinically classified intraoperatively as phlegmonous appendicitis were subsequently confirmed as NGA on histology, highlighting the limitations of macroscopic assessment alone. Díaz et al. [28] showed that standardized postoperative protocols in pediatric gangrenous appendicitis reduced intra-abdominal abscess (IAA) rates and hospital stays. Although their study focused on macroscopic diagnosis, their results align with ours, suggesting that SDAT is safe and effective. Also, in a randomized trial by Eddine et al. [29], most patients with clinically confirmed gangrenous appendicitis received short-course antibiotics, despite their classification as complicated. Similarly, 72% of clinically diagnosed NGA patients received SDAT per guidelines, while 28% received POAT based on clinical evaluation. Notably, hospital stays remained shorter than those reported in studies involving prolonged antibiotic use.

This study should be interpreted in light of its limitations. The retrospective, single-centre design introduces a risk of selection bias and limits generalizability. Antibiotic treatment was determined by the operating surgeon’s clinical judgement rather than by a standardized treatment protocol, resulting in numerical imbalance and baseline differences between the SDAT and POAT groups. Furthermore, the modest sample size and low number of postoperative surgical infectious complications limited statistical power and precluded robust multivariable adjustment. Although propensity score matching reduced measured baseline imbalance, residual confounding from unmeasured clinical factors cannot be excluded. Nevertheless, the absence of worse infectious outcomes among matched patients treated with SDAT supports the interpretation that postoperative antibiotics may not be routinely required in non-perforated gangrenous appendicitis. Prospective, multicentre studies with standardised antibiotic protocols are warranted to confirm these findings.

## Conclusion

Among adults with non-perforated gangrenous appendicitis treated according to surgeon judgement, single-dose antibiotic therapy was not associated with higher rates of early postoperative infectious complications compared with postoperative antibiotic therapy. Although patients selected for POAT appeared to have greater baseline clinical severity, propensity score–matched analysis showed comparable infectious outcomes between treatment strategies. These findings suggest that SDAT may be a sufficient antibiotic strategy for this patient group and that routine postoperative antibiotics may not provide additional benefit in uncomplicated, non-perforated gangrenous appendicitis. Given the retrospective design and potential for residual confounding, these results should be interpreted as associations observed in real-world practice rather than evidence of causality. Prospective, multicentre studies with standardized antibiotic protocols are warranted to confirm whether shorter antibiotic courses can be adopted safely.

## Supplementary Information

Below is the link to the electronic supplementary material.

Supplementary file1 (DOCX 33 KB)

## Data Availability

No datasets were generated or analysed during the current study.
