# Supplementary material for: Antibiotic stewardship in non-perforated gangrenous appendicitis: outcomes of single-dose versus postoperative therapy in a retrospective cohort study
Source: Langenbecks Arch Surg. 2026 Jul 3;411(1):261. doi: 10.1007/s00423-026-04129-9 (PMC13633304; doi:10.1007/s00423-026-04129-9)
Supplement: Supplementary file 1 [file 423_2026_4129_MOESM1_ESM.docx]

| **Supplementary Table S1.** Multivariable Logistic Regression Analysis of Factors Associated with Selection of Postoperative Antibiotic Therapy (POAT) | | | |
| --- | --- | --- | --- |
|  | Adjusted OR | 95% CI | P-value |
| Age (per year increase) | 0.98 | 0.96-1.00 | **0.032** |
| ASA Score (per 1‑point increase) | 0.81 | 0.49-1.33 | 0.408 |
| CRP at admission (mg/L) | 0.99 | 0.98-0.99 | <**0.001** |
| q-SOFA Score (per 1‑point increase) | 0.30 | 0.11-0.82 | **0.018** |
| Alvarado Score (per 1‑point increase) | 1.05 | 0.89-1.25 | 0.539 |
| Abbreviations: NGA; non-perforated gangrenous appendicitis, ASA; American Society of Anesthesiologists; q-SOFA; quick Sepsis-related Organ Failure Assessment, SDAT; single-dose antibiotic therapy, POAT; postoperative antibiotic therapy, OR; odds ratio  POAT administration was independently associated with age, CRP, and q‑SOFA, indicating that postoperative antibiotic therapy tended to be reserved for patients perceived as having higher inflammatory and systemic severity. | | | |
